# The *INSIG2 *rs7566605 polymorphism is not associated with body mass index and breast cancer risk

**DOI:** 10.1186/1471-2407-10-563

**Published:** 2010-10-18

**Authors:** Daniele Campa, Anika Hüsing, James D McKay, Olga Sinilnikova, Ulla Vogel, Anne Tjønneland, Kim Overvad, Jakob Stegger, Françoise Clavel-Chapelon, Nathalie Chabbert-Buffet, Guy Fagherazzi, Antonia Trichopoulou, Dimosthenis Zylis, Erifili Oustoglou, Sabine Rohrmann, Birgit Teucher, Eva Fisher, Heiner Boeing, Giovanna Masala, Vittorio Krogh, Carlotta Sacerdote, Salvatore Panico, Rosario Tumino, N Charlotte Onland-Moret, Carla H van Gils, H Bas Bueno-de-Mesquita, Eiliv Lund, María Dolores Chirlaque, Núria Sala, José Ramon Quirós, Eva Ardanaz, Pilar Amiano, Esther Molina-Montes, Göran Hallmans, Per Lenner, Ruth C Travis, Timothy J Key, Nick Wareham, Kay-Tee Khaw, Sabina Rinaldi, Nadia Slimani, Veronique Chajes, Afshan Siddiq, Elio Riboli, Rudolf Kaaks, Federico Canzian

**Affiliations:** 1 German Cancer Research Center (DKFZ), Heidelberg, Germany; 2 International Agency for Research on Cancer, Lyon, France; 3 Hospices Civils de Lyon/Centre Léon Bérard, UMR5201 CNRS-Université Claude Bernard, Lyon, France; 4 National Food Institute Technical University of Denmark, Denmark; 5 Danish Cancer Society, Copenhagen, Denmark; 6 Aarhus University Hospital, Aalborg, Denmark; 7 Department of Cardiology, Cardiovascular Research Centre, Aalborg Hospital, Aarhus University Hospital, Aalborg, Denmark; 8 Institut Gustave Roussy, Villejuif, France; 9 WHO Collaborating Center for Food and Nutrition Policies, Department of Hygiene, Epidemiology and Medical Statistics, University of Athens Medical School, Athens, Greece; 10 Hellenic Health Foundation, Athens, Greece; 11 German Institute of Human Nutrition, Potsdam-Rehbruecke, Germany; 12 Molecular and Nutritional Epidemiology Unit, Cancer Research and Prevention Institute (ISPO), Florence, Italy; 13 Istituto Nazionale dei Tumori (IRCCS), Milan, Italy; 14 CPO Piemonte, Turin, Italy; 15 Department of Clinical and Experimental Medicine Federico II University, Naples, Italy; 16 Azienda Ospedaliera "Civile M.P.Arezzo" Ragusa, Italy; 17 Julius Center, University Medical Center, Utrecht, The Netherlands; 18 National Institute for Public Health and the Environment, Bilthoven, The Netherlands; 19 Institute of Community Medicine University of Tromsø, Tromsø, Norway; 20 Murcia Regional Health Council, Murcia, Spain; 21 Catalan Institute of Oncology, Barcelona, Spain; 22 Consejería de Salud y Servicios Sanitarios Principado de Asturias, Oviedo, Spain; 23 Public Health Institute of Navarra, Pamplona, Spain; 24 Public Health Department of Gipuzkoa, San Sebastian, Spain; 25 Andalusian School of Public Health, Granada, Spain; 26 CIBER de Epidemiología y Salud Pública (CIBERESP), Spain; 27 Umeå University, Umeå, Sweden; 28 Cancer Epidemiology Unit, Nuffield Department of Clinical Medicine, University of Oxford, Oxford, UK OX3 7LF; 29 University of Cambridge, Cambridge, UK; 30 Imperial College, London, UK

## Abstract

**Background:**

The single nucleotide polymorphism rs7566605, located in the promoter of the *INSIG2 *gene, has been the subject of a strong scientific effort aimed to elucidate its possible association with body mass index (BMI). The first report showing that rs7566605 could be associated with body fatness was a genome-wide association study (GWAS) which used BMI as the primary phenotype. Many follow-up studies sought to validate the association of rs7566605 with various markers of obesity, with several publications reporting inconsistent findings. BMI is considered to be one of the measures of choice to evaluate body fatness and there is evidence that body fatness is related with an increased risk of breast cancer (BC).

**Methods:**

we tested in a large-scale association study (3,973 women, including 1,269 invasive BC cases and 2,194 controls), nested within the EPIC cohort, the involvement of rs7566605 as predictor of BMI and BC risk.

**Results and Conclusions:**

In this study we were not able to find any statistically significant association between this SNP and BMI, nor did we find any significant association between the SNP and an increased risk of breast cancer overall and by subgroups of age, or menopausal status.

## Background

Herbert and coworkers [1] conducted a genome-wide association study (GWAS) on 694 subjects of the Framingham Heart Study with a family-based design to identify genetic loci (single nucleotide polymorphisms, SNPs) contributing to obesity using body mass index (BMI) as the primary phenotype. The authors reported strong statistical evidence that the SNP rs7566605 located near the 5' end of *INSIG2 *(Chr2:118,552,255) was associated with increased BMI [1]. In this initial study, association of rs7566605 with BMI was replicated in ethnically distinct populations, including European and African Americans. *INSIG2 *has been functionally linked to lipid metabolism, due to its role in endogenous cholesterol and fatty acid synthesis feedback inhibition [2]. It is an endoplasmic reticulum membrane bound protein that inhibits the proteolytic activation of Sterol Response Element Binding Proteins (SREPs) in response to cholesterol or insulin [2]. Thus, the biological function of the *INSIG2 *gene product is consistent with the initially observed association.

A series of follow-up studies sought to validate the association of *INSIG2 *rs7566605 with markers of obesity in humans, with several publications reporting inconsistent findings [3-13].

There is ample evidence that excessive body weight is a risk factor for a number of diseases. In particular, the evidence that body fatness is a cause of postmenopausal breast cancer (BC) is convincing [14]. On the other hand, body fatness is inversely associated with BC risk in premenopausal women [14].

Body fatness directly affects levels of many circulating hormones, such as insulin, insulin-like growth factors, and oestrogens, creating an environment that favors carcinogenesis and hinders apoptosis (WCRF/AICR, 2007). It also stimulates the body's inflammatory response, which may contribute to the initiation and progression of several cancers (WCRF/AICR, 2007).

We tested the SNP association with the body-mass index (BMI) and with BC risk in a large study of 3,973 women nested within the European Prospective Investigation into Cancer and Nutrition (EPIC).

## Methods

### The EPIC cohort

A fully detailed description of the EPIC cohort has been published elsewhere [15]. Briefly, EPIC consists of about 370,000 women and 150,000 men, aged 35-69, recruited between 1992 and 2005 in 10 Western European countries. The vast majority (>97%) of subjects recruited in the EPIC cohort are of European ('Caucasian') origin. All EPIC study subjects provided anthropometric measurements (height, weight, and waist and hip circumferences) and extensive, standardized questionnaire information about medical history, diet, physical activity, smoking, and other lifestyle factors. Women also answered questions about menstrual and reproductive history, hysterectomy, ovariectomy, and use of exogenous hormones for contraception or treatment of menopausal symptoms. Based on the questionnaire data, 32% of the subjects were pre-menopausal at blood donation, 10% were peri-menopausal or of unknown menopausal status, and 58% were post-menopausal. About 260,000 women and 140,000 men provided a blood sample. Cases of cancer occurring after recruitment into the cohort and blood donation are identified through local and national cancer registries in 7 of the 10 countries, and in France, Germany, and Greece by a combination of contacts with national health insurances and/or active follow-up through the study subjects or their next of kin. Follow-up on vital status is achieved through record linkage with mortality registries.

### Selection of case and control subjects

Case subjects were selected among women who developed BC after blood collection. Control subjects (1-2 controls per case) were selected randomly by incidence density sampling, matching the cases for centre of recruitment, age at blood donation, duration of follow-up, menopausal status at the time of blood donation and use of exogenous hormones. This study did not include women who were using hormone replacement therapy (HRT) at the time of blood donation. For the present study on BMI, 3,973 unique subjects were considered, of whom 1,269 invasive BC cases and 2,194 controls were included in the BC risk analysis. Subjects who were not included in the breast cancer risk analysis consisted of 108 cases with carcinomas *in situ *and 206 matched controls, and 123 cases (whose matched controls had genotyping failure) and 125 controls (whose matched cases had genotyping failure). Incidence density matching was applied, resulting in the selection of 52 subjects as duplicate controls for conditional analyses. Each control should have been free of cancer up to the duration of follow-up of the index case. All participants signed an informed written consent and the study was approved by the ethical review boards of the International Agency for Research on Cancer, and of the collaborating institutions responsible for subject recruitment in each of the EPIC recruitment centres.

### DNA extraction and genotyping

DNA was extracted from blood samples on an Autopure instrument (Qiagen, Hilden, Germany) with Puregene chemistry (Qiagen, Hilden, Germany). The order of DNAs from cases and controls was randomized on PCR plates in order to ensure that an equal number of cases and controls could be analyzed simultaneously. All the genotyping was carried out using the Taqman assay (Applied Biosystem, Foster City, California, USA). Details on the genotype procedure are described elsewhere [16]. All samples that did not give a reliable result in the first round of genotyping were resubmitted to up to two additional rounds of genotyping. Data points that were still not filled after this procedure were left blank. Repeated quality control genotypes (8% of the total) showed a concordance of 99.7%.

### Statistical analysis

The frequency distribution of genotypes was examined for cases and controls and deviation of genotype frequencies from the Hardy-Weinberg equilibrium was assessed in the controls by chi-square test. Incidence density matching was used to match controls to cases, resulting in 52 controls being selected twice into 4,025 study subjects with genotypes. We used conditional logistic regression for multivariate analyses to assess the main effects of the genetic polymorphism on BC risk assuming codominant model of inheritance. The more common allele in the controls (C allele) was held as the reference category in calculating the odds ratios (OR). All relative risk analysis were performed with the exclusion of cases with carcinomas *in situ *(n = 108 cases and 206 matched controls). For this analysis incomplete case-control sets could not be used and therefore 248 subjects were excluded. Subgroup analyses were performed based on menopausal status at recruitment and age at recruitment or diagnosis (with cutpoint at 50 or 55 years of age at diagnosis. Due to the lack of the information on the menopausal status at diagnosis, age at diagnosis can be used as a surrogate. In particular, we can assume that all women older than 55 at diagnosis were in menopause). Additional analyses were performed by including cases of carcinoma *in situ*, and by excluding cases diagnosed shortly (6 months, 1 or 2 years) after blood drawing.

The association between the SNP and BMI was estimated by unconditional regression models, and weighted means in each genotype category were calculated adjusting for age and case-control-status. For this analysis we could use the genotypes of all 3,973 unique subjects subjects, regardless of case-control and matching status.

A possible association between the SNP and BMI was investigated also with unconditional logistic regression calculating odds ratios for genotype trend models nested within categories of BMI as below 25 (normal weight), 25 ≤ BMI <30 (overweight) and BMI ≥ 30 (obese). This model was compared to a main effects model with a likelihood ratio test of 2 degrees of freedom.

## Results

We explored the association between a SNP in the *INSIG2 *gene promoter (rs7566605, Chr2:118,552,255) and BMI, as well as with BC risk. We included 1,269 incident BC cases from the EPIC cohort and 2,194 matched controls for the risk and 3,973 subjects for the BMI analysis. Table 1 summarizes the baseline characteristics of cases and controls. The genotype distribution was in Hardy-Weinberg equilibrium in controls, with a non-significant chi square value (data not shown). The frequencies and distribution of the genotypes and the odds ratios for the association with BMI and BC risk are described in Table 2.

**Table 1:** Baseline characteristics of BC cases and controls.

Variable	Cases	Controls*
Subjects with genotypes	1,500	2,473

Women with carcinoma *in situ*	108^a^	206^a^

Incomplete matched sets	123	125

Complete match-sets excluding carcinoma *in situ*	1,269	2,194

Pre-menopausal women	388	738
Peri-menopausal women	138	221
Post-menopausal women	974	1,514

Mean age at blood donation	55.2 (40.7-67.5)^b^	54.8 (40.1-68.1)^b^
Mean age at diagnosis	57.5 (43.5-70.0)^b^	-
Height	161.4 (151.0-172.0)^b^	160.7 (150.0-172.0)^b^
Weight	67.6 (51.7-89.0)^b^	67.3 (51.0-89.5)^b^
Body mass index	26.0 (20.3-34.5)^b^	26.1 (20.1-35.0)^b^

^a ^Subjects with carcinomas *in situ *and their matched controls were not included in the BC risk analysis

^b ^Mean (5th - 95th percentiles)

*52 duplicated controls are included in this total

**Table 2:** Association of rs7566605 with BMI and BC risk.

rs7566605	CC	GC	GG	P-values
Cases	562	572	135	
Controls	944	1,008	242	
OR ^a ^(BC risk)	1	0.95 (0.82-1.10)	0.93 (0.74-1.19)	0.466^c^

Normal weight (BMI <25)	797	862	208	
Overweight (BMI <30)	656	624	143	
Obese (BMI ≥30)	295	308	80	
BMI^b^	26.08 (25.87-26.30)	26.00 (25.78-26.21)	26.05 (25.62-26.48)	0.697^c^
OR overweight or obese^d^	1	0.90 (0.79-1.03)	0.88 (0.71-1.08)	0.104^c^
OR obese^d^	1	0.95 (0.79-1.15)	1.00 (0.75-1.34)	0.823^c^

^a^OR: odds ratio for BC risk from conditional logistic regression analysis on matched data; cases of carcinoma *in situ *and their controls were excluded from the analysis, the C allele was taken as reference.

^b^Body Mass Index. Adjusted for age and case-control status; cases of carcinoma *in situ *and their controls were included in the analysis, mean (5th - 95th percentiles)

^c^P test of trend

^d^OR: odds ratio from unconditional logistic regression comparing this outcome to normal weight, adjusted for age and case-control-status. The C allele was taken as reference.

We observed no statistically significant association between the SNP and the geometric means of BMI. We performed an additional analysis by categorizing subjects with BMI ≥ 30 as obese, 25 ≤BMI <30 as overweight and <25 as normal weight, and calculating odds ratios for obese or overweight versus normal weight with unconditional logistic regression, but we found no statistically significant association. We performed the analysis considering the cases and the controls separate or together and the results obtained did not change. We have also stratified for menopausal status at baseline and we did not find any difference in the genotype distribution. (data not shown).

We performed also an analysis of BC risk. We found no statistically significant associations with risk overall and stratified by age or menopausal status, with codominant model. Additional analyses performed by including cases of carcinoma *in situ*, or excluding cases diagnosed shortly after blood drawing, showed essentially the same results as using all cases and controls (data not shown).

## Discussion

In this report we explored the possible association between a SNP in the *INSIG2 *gene and BMI and with BC risk in a large population from the EPIC cohort.

In recent years the search for genetic factors predisposing to obesity has intensified. Several candidate and GWAS have been performed, and have led to the identification of a number of common common genetic variants related to obesity [[6,17-19], Walley, 2009 #167, [20]].

The *INSIG2 *gene is a good candidate for being related to obesity because of its function in lipid metabolism, particularly in blocking the processing of SREBPs in response to cholesterol or insulin. A SNP lying approximately 10 kb upstream from *INSIG2*, and thus potentially affecting the transcriptional activity of the gene, rs7566605, has been found to be associated with BMI in a family-based GWAS [1]. Replication of this result has been attempted in multiple studies, with some studies confirming an association [3,4,6-13,21] and some others not finding it [3,4,6-13,21]. Recently a meta-analysis has been published on the association of this SNP in *INSIG2 *and obesity [22] The results of the study from Heid and collaborators suggests that the association is potentially valid but also suggests the possibility that heterogeneity could hinder possible findings if not taken into account properly.

Thus, evidence for or against the association of this SNP with obesity is still needed. We sought to provide it, at least for the Caucasian population, with a study nested within the EPIC cohort. EPIC is an ideal setting, since it has a large population size, accurate phenotypic information on BMI for all subjects, and the presence of both pre-menopausal and post-menopausal women. We tested the effect of rs7566605 on BMI and, at the same time, on BC risk.

We exhaustively analyzed the possible associations between the SNP, BMI and BC risk.. Moreover, subgroup analyses were performed based on menopausal status and age. We have found no statistical association between the polymorphism and the two selected end-points.

We had greater than 99% power to replicate the association of the SNP with BMI, and greater than 80% power to detect an association between rs7566605 and BC risk, with an odds ratio of at least 1.3 and a type 1 error of 0.05.

A possible limitation of this report could be the fact the majority of women and BC cases included in the study are post-menopausal. The power among pre-menopausal women was of 0.81 to detect an OR of 1.3 in a log additive model, hence we cannot exclude a minor effect of the variant allele on BC risk in pre-menopausal subjects. Moreover a possible explanation for a lack of association of this polymorphism with the two endpoints could be the role of gene-environment interactions.

## Conclusions

In conclusion, we have studied association of the polymorphism rs7566605, located near the 5' end of the *INSIG2 *gene, with BMI and BC risk within the EPIC study and we can confidently exclude a major role of this polymorphism with respect to both end-points in this population.

## Competing interests

The authors declare that they have no competing interests.

## Authors' contributions

RK, FC conceived and designed the experiments, DC performed the experiments AH analyzed the data: DC, FC, AH, JDMK, OS, RK drafted the manuscript. All other authors contributed substantially to sample collection and manuscript editing.

All authors read and approved the final version of the manuscript.

## Pre-publication history

The pre-publication history for this paper can be accessed here:

http://www.biomedcentral.com/1471-2407/10/563/prepub
